# Genome-Wide Characterization and Seasonal–Circadian Expression Analysis of CCT Family Genes in *Populus*

**DOI:** 10.3390/genes17030346

**Published:** 2026-03-20

**Authors:** Rui Zang, Yue Li, Xiaokang Dai

**Affiliations:** 1 Engineering Research Center of Agricultural Microbiology Technology, Ministry of Education & Heilongjiang Provincial Key Laboratory of Ecological Restoration and Resource Utilization for Cold Region & Key Laboratory of Microbiology, College of Heilongjiang Province & School of Life Sciences, Heilongjiang University, Harbin 150080, China; 2 Hubei Hongshan Laboratory, Hubei Engineering Technology Research Center for Forestry Information, College of Horticulture and Forestry Sciences, Huazhong Agricultural University, Wuhan 430070, China

**Keywords:** *Populus*, CCT gene family, seasonal dormancy

## Abstract

Background: The CONSTANS, CONSTANS-like, and TIMING OF CAB EXPRESSION 1 (CCT) domain proteins are key regulators of flowering time and circadian rhythms in annual plants, but their diversity and temporal expression patterns in perennial trees remain poorly understood. Methods: Here, we performed a genome-wide characterization of CCT family genes and analyzed their seasonal and circadian expression dynamics in *Populus*. Using an HMM-based search, we identified 49 putative CCT genes (*PtCCTs*) in the *Populus* genome and classified them into five subfamilies (COL, CMF, PRR, ALSM and ZIM) based on domain composition and phylogeny. Results: Synteny and duplication analyses showed that most *PtCCTs* arose from segmental duplication and have predominantly evolved under purifying selection. Promoter analyses revealed a rich repertoire of cis-regulatory elements, with a marked enrichment of light- and hormone-responsive motifs, particularly G-box and ABRE elements, in *PtPRR* and a subset of *PtCOL* promoters. Transcriptome data indicated that many *PtCCTs* display distinct tissue-specific expression patterns, with *PtPRRs* and *PtZIMs* being strongly enriched in dormant buds. Seasonal transcriptomes from leaves and shoot apices revealed discrete expression profiles associated with growth, bud set, and winter dormancy, and most *PtPRRs* showed increasing transcript levels from September to December. Diurnal time-series data further identified 19 *PtCCTs* with significant rhythmic expression, separating COL and PRR members into night- and day-phased groups. Network analysis using STRING indicated that *PtPRRs* interact with photoperiodic pathway components such as *PtGI*, and re-analysis of diurnal data from wild-type and lhy-RNAi hybrid aspen showed that several *PtPRRs* exhibit phase and amplitude changes when LHY expression is reduced. Conclusions: Together, these results provide a comprehensive overview of the CCT gene family in *Populus* and highlight *PtPRRs* and specific *PtCOLs* as promising candidates that link the circadian clock and light signaling to seasonal growth cessation and bud dormancy in perennial trees.

## 1. Introduction

The CCT family genes (*CONSTANS* (*CO*), *CONSTANS-like* (*COL*), and *timing of CAB expression1* (*TOC1*)) contain a conserved motif (CCT domain, PF06203) of ~43 amino acid residues [1]. These genes are recognized for their pivotal roles in orchestrating circadian rhythms and bolstering plant resilience to abiotic stresses, thereby exerting profound influence over various facets of plant growth, development, and flowering processes [2]. The CCT family genes can be divided into several categories according to their conserved domains [3]: *COL*, *CMF* (CCT Motif Family), *PRR* (Pseudo-Response Regulator), and *ZIM* (Zinc-finger Inflorescence Meristem). *COL* genes, distinguished by the presence of one or two B-box domains in addition to the CCT domain, have been discerned across diverse plant species [4,5,6,7]. Notably, COL genes exert significant influence over the regulation of flowering, particularly in response to light-mediated cues [8]. The CMF encodes proteins possessing only one CCT domain. In *Arabidopsis*, *CIA2* (*CHLOROPLAST IMPORT APPARATUS 2*) and its homolog *CIL* (*CIA2-like*) are instrumental in chloroplast translation and play a crucial role in modulating responses to UV-AB, high light exposure, and heat shock [9]. Meanwhile, *ALSM2 (Activator of Spo^min^::LUC2)* can regulate the expression of a subset of sugar-inducible genes in *Arabidopsis* [10]. Notably, the rice CMF gene *Ghd7* (Grain number, plant height, and heading date 7) exerts a marked effect on stem growth and development, thus assuming a pivotal role in augmenting seed yield [11]. Within the PRR subfamily, genes are functionally conserved in regulating circadian clock output pathways [12]. All the *PRR* genes (*AtPRR9*, *AtPRR7*, *AtPRR5*, *AtPRR3*, and *AtTOC1*) in *Arabidopsis* are regulated by the circadian clock [13]. The *ZIM* sub-family genes contain a TIFY domain, a CCT domain, and a GATA zinc-finger domain [14]. Within the *ZIM* subfamily, *Arabidopsis ZIM LIKE1/2* (*ZML1* and *ZML2*) are involved in cryptochrome 1-dependent responses to excess light [15], and the *MYB*/*ZML* complex (*MYB11* and *ZML2*) is involved in wound-induced lignification in maize [16]. The growing availability of genomic resources has facilitated the systematic identification of CCT family genes in various plant species, enabling comprehensive investigations into their functions [17,18,19].

Perennial woody trees inhabiting temperate and boreal regions have developed an intricate activity–dormancy cycle, a pivotal adaptive trait crucial for their survival and growth [20,21,22]. To endure the harsh winter conditions prevalent in these regions, many of these trees enter a state of dormancy by suppressing active growth, typically coinciding with leaf senescence and abscission [22]. The regulation of this activity–dormancy cycle in perennial woody trees is generally contingent upon key environmental cues such as photoperiod and temperature [20]. *Populus*, an exemplary species for the study of seasonal activity–dormancy cycles, predominantly thrives in the temperate and boreal zones, where it employs dormancy as an adaptive strategy to confront inclement winter conditions. In *Populus*, a specific subset of the CCT family genes, particularly COL and PRR genes, have been identified in previous studies as pivotal contributors to circadian rhythms [8,23]. Specifically, *PtCO2* has been empirically demonstrated to regulate growth cessation and bud set in poplar [4]. The *CO*/*FT* module, comprising *PtCO2* and *PtFT1*, not only exerts control over flowering time in aspen trees but also governs growth cessation and bud set during the autumn [4]. In addition, the *PtPRR5* gene exhibits distinct expression patterns, upregulating when transitioning from long days (LDs) to short days (SDs) and subsequently downregulating during the bud break periods [24,25], thereby implying a potential role of *PtPRR5* in influencing growth cessation and bud set in *Populus*. At the gene family level, a recent genome-wide analysis in poplar identified 39 CCT transcription factors, characterized their structures, chromosomal distribution, synteny relationships, and promoter cis-elements, and functionally showed that *PpnCCT39* enhances chlorophyll content and photosynthetic rate when overexpressed in poplar [26]. This work provided an important foundation for CCT family studies in poplar but mainly focused on gene family structure and a photosynthesis-related function of a single member. In contrast, how the CCT gene family as a whole is deployed in perennial tissues across seasonal transitions and how it is integrated into circadian regulation and dormancy control remain poorly understood.

In this study, we performed a genome-wide analysis of CCT family genes in *Populus*, including gene structure, phylogeny, synteny and gene duplication, cis-regulatory elements, and tissue, seasonal and diurnal expression patterns. By integrating RNA-seq datasets from multiple tissues, year-round seasonal time-series in leaves and shoot apices, and diurnal expression profiles, we provide a comprehensive overview of *PtCCTs* and highlight *PtPRRs* and specific *PtCOLs* as promising candidates linking the circadian clock and light signaling to seasonal growth cessation and bud dormancy in *Populus*.

## 2. Materials and Methods

### 2.1. Identification of CCT Genes in Populus

To identify the CCT family genes in *Populus*, the hidden Markov model (HMM) profile of the CCT domain (PF06203) was downloaded from the PFAM database [27] and searched against all protein sequences in *P. trichocarpa* v3.1 with hmmsearch v3.4 [28] with a threshold e-value < 10 × 10^−10^. After the initial search, redundant candidate sequences were removed based on duplicated protein entries and gene IDs in the genome annotation. The remaining non-redundant candidate proteins were then examined for the presence of the CCT domain using the PFAM database and further validated with InterProScan and SMART. Only sequences confirmed to contain a complete CCT domain were retained for subsequent analyses. Using the same procedure, CCT family genes were also identified in *Arabidopsis thaliana*, and *CCT* gene lists for rice were retrieved from a previous study [29]. The physicochemical properties of PtCCT proteins, including molecular weight (MW), theoretical isoelectric point (pI), instability index (II), and grand average of hydropathicity (GRAVY), were calculated using the ProtParam tool on the ExPASy server (http://www.expasy.org/). To characterize domain compositions of the Populus CCT family, all CCT protein sequences were further scanned against PFAM with hmmsearch using the same E-value threshold. Gene structures were subsequently visualized with TBtools TBtools [30].

### 2.2. Phylogenetic, Synteny, and Cis-Element Analyses

Maximum likelihood (ML) phylogenetic trees were constructed using RAxML v8.2.12 [31] with 1000 bootstrap replicates under the PROTGAMMALGX amino acid substitution model and were visualized in FigTree. Collinearity and gene duplication of CCT family genes were analyzed with MCScanX v1.0.0 [32], and syntenic relationships were displayed using Circos v0.69 [33]. Nonsynonymous (*Ka*) and synonymous (*Ks*) substitution rates were estimated with TBtools [30]. The timing of gene duplication events was inferred from *Ks* values, employing the formula T = *Ks*/2λ, with a λ value of 9.1 × 10^−9^ being considered for *Populus* [34]. *Cis*-regulatory elements within the 2 kb upstream regions of coding sequences (CDSs) were identified using the PlantCARE web database [35] and subsequently visualized with TBtools [30].

### 2.3. Expression Pattern Analysis of CCT Family Genes in Populus

To characterize expression patterns of CCT genes in *Populus*, we integrated several publicly available RNA-seq datasets. Tissue-specific expression of *PtCCTs* was analyzed using RNA-seq data from the PhytoMine database for female flowers (FF), male flowers (MF), pre-dormant buds (PD), early dormant buds (EDB), late dormant buds (LDB), fully open buds, fully expanded leaves (EL), young leaves (YL), immature leaves (IL), root tips (RT), roots (R), and stems (S).

Seasonal expression patterns were further examined using leaf transcriptome data spanning May to October in 2015 and 2016 (BioProject PRJNA597006 [36]), together with vegetative shoot apex (SA) transcriptome data from our previous study covering September to May [37]. Read counts from these datasets were normalized to reads per kilobase per million mapped reads (RPKM), and genes with distinct seasonal expression profiles in SA and leaf tissues were identified using Short Time-series Expression Miner (STEM) [38], with the maximum number of model profiles set to 20 and all other parameters kept at their default values. Only significantly enriched model profiles with *p* < 0.05 were considered significant.

Diurnal dynamics were analyzed using a diurnal expression matrix from hybrid aspen (*Populus tremula* × *P. tremuloides*; clone T89) reported by Edwards et al. [39]. Diurnally rhythmic PtCCTs were identified using the JTK_CYCLE method [40] implemented in the MetaCycle (v1.2.0) R package with the meta2d function [41]; genes with JTK_pvalue < 0.05 were considered diurnally oscillating. Because JTK_pvalue was used as the significance criterion, this term is explicitly retained throughout the manuscript to avoid confusion with adjusted *p* values from other methods.

## 3. Results

### 3.1. Genome-Wide Identification of CCT Family Genes in Populus

**Figure 1:**
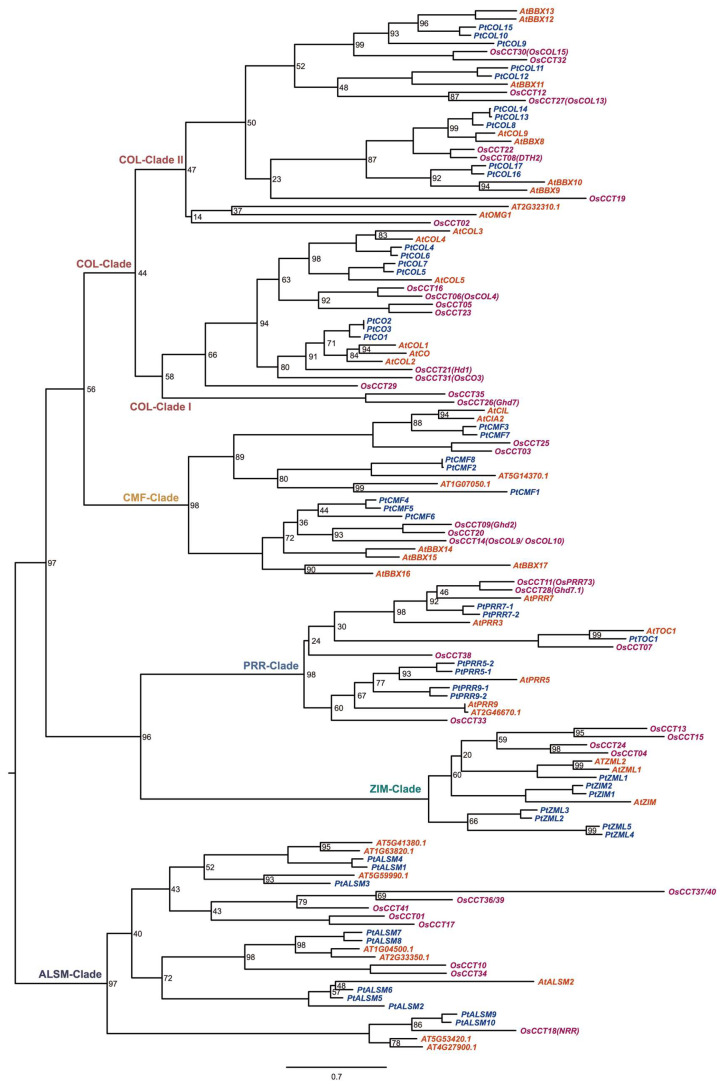
Phylogenetic tree of CCT family genes in *Populus*, rice, and *Arabidopsis*. A maximum likelihood phylogenetic tree was constructed using RAxML with 1000 bootstrap replicates under the PROTGAMMALGX amino acid substitution model and visualized with FigTree. CCT genes from *Populus*, rice, and *Arabidopsis* are shown in blue, purple, and red, respectively. The major clades are indicated by colored labels, including the COL-clade (CONSTANS-like), which is further divided into COL-Clade I and COL-Clade II, as well as the CMF-clade (CCT Motif Family), PRR-clade (Pseudo-Response Regulator), ZIM-clade (Zinc-finger Inflorescence Meristem), and ALSM-clade (Activator of Spomin::LUC2-like). Bootstrap values are shown on the branches, except for branches with a bootstrap value of 100.

**Figure 2:**
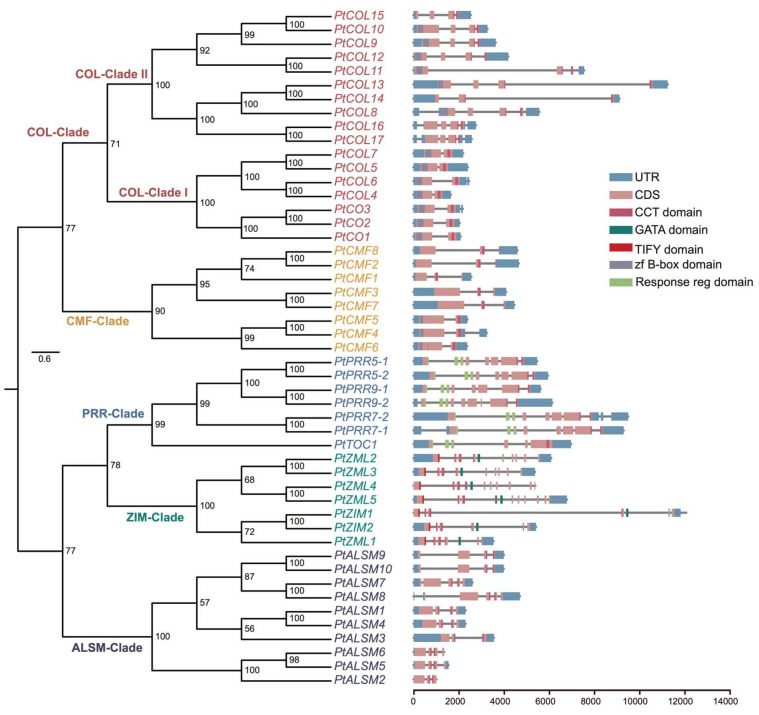
Phylogenetic relationships and gene structures of PtCCT family genes. A maximum likelihood phylogenetic tree of *PtCCT* genes was constructed using RAxML with the same parameters as described for Figure 1. The major clades are indicated by colored labels: COL-clade (CONSTANS-like), including COL-Clade I and COL-Clade II; CMF-clade (CCT Motif Family); PRR-clade (Pseudo-Response Regulator); ZIM-clade (Zinc-finger Inflorescence Meristem); and ALSM-clade (Activator of Spomin::LUC2-like). Bootstrap values are shown on the branches. The gene structures and conserved domains of *PtCCT* genes are shown on the right. Blue boxes represent untranslated regions (UTRs), pink boxes represent coding sequences (CDSs), and gray lines represent introns. Conserved domains are indicated by colored boxes corresponding to the legend.

Based on the HMM-based profile, we identified a total of 49 putative *PtCCTs* and subsequently categorized them into five distinct groups (COL, CMF, PRR, ZIM, and ALSM) based on the outcome of gene domain compositions and the resulting phylogenetic tree (Figure 1 and Figure 2). Specifically, we discerned 17 putative *PtCCTs*, termed CO-like (*COL*) genes, with 13 out of the 17 genes containing two zf B-box domains. These *COL* genes were further subdivided into two distinct subclades. Additionally, we observed genes that exclusively featured a single CCT motif and noted that they clustered within two separate clades: the CMF and ALSM clades. Notably, the CMF clade exhibited proximity to the COL clade, and we designated these eight genes as *PtCMFs*. Conversely, the ALSM clade shared a clustering pattern with the *AtASML2* gene, known for its role in regulating the expression of a subset of sugar-inducible genes [10]. This clade encompassed 10 *PtALSM* genes. Within the PRR clade, we identified seven *PtPRR* genes, which included two *PtPRR5s*, two *PtPRR7s*, two *PtPRR9s*, and one *PtTOC1*. These genes were characterized by the presence of a response-regulator domain and a CCT motif. Lastly, we observed the presence of seven *PtZIM* genes, each featuring a GATA, TIFY, and CCT domain. Comprehensive statistical information detailing the predicted CCT proteins can be found in Table S1.

Furthermore, the cis-regulatory elements of *PtCCT* genes were predicted from 2 kb upstream sequences of CDSs using PlantCARE [35]. In total, our analysis yielded 7984 cis-regulatory elements, representing 99 different types, within the 2-kilobase promoter regions. Building upon existing knowledge of their functions, we subsequently categorized these identified cis-elements into four distinct groups, encompassing developmental, hormonal response, light response, and stress response categories. Our findings underscored a pronounced enrichment of cis-regulatory elements in the hormonal and light response categories, with particular emphasis on *PtPRRs* and a subset of *PtCOLs* (Figure S1).

### 3.2. Synteny Analyses and Gene Duplication

The 49 identified *PtCCT* genes displayed an uneven distribution across 14 of the 19 chromosomes and 3 scaffolds in the *Populus* genome. Notably, within this gene set, 20 gene pairs were indicative of segmental duplications, whereas only one gene pair was suggestive of tandem duplication, as delineated in Figure S2 and detailed in Table S2. The occurrences of segmental duplication events hold particular significance in facilitating the expansion of the CCT family genes within *Populus*. Furthermore, we computed the *Ka* and *Ks* values for each pair of duplicated genes. Notably, two distinct *Ks* ranges were identified (Table S3): 0.2061–0.3658, encompassing 16 gene pairs, and 1.499–2.339, which applied to five gene pairs. Additionally, we assessed the *Ka*/*Ks* ratios for these duplicated gene pairs, which spanned from 0.1187 to 0.4392, with a mean value of 0.2808. This observation underscores that the CCT family genes within *Populus* primarily underwent purifying selection.

### 3.3. Transcriptome Expression of PtCCTs in Different Tissues

**Figure 3:**
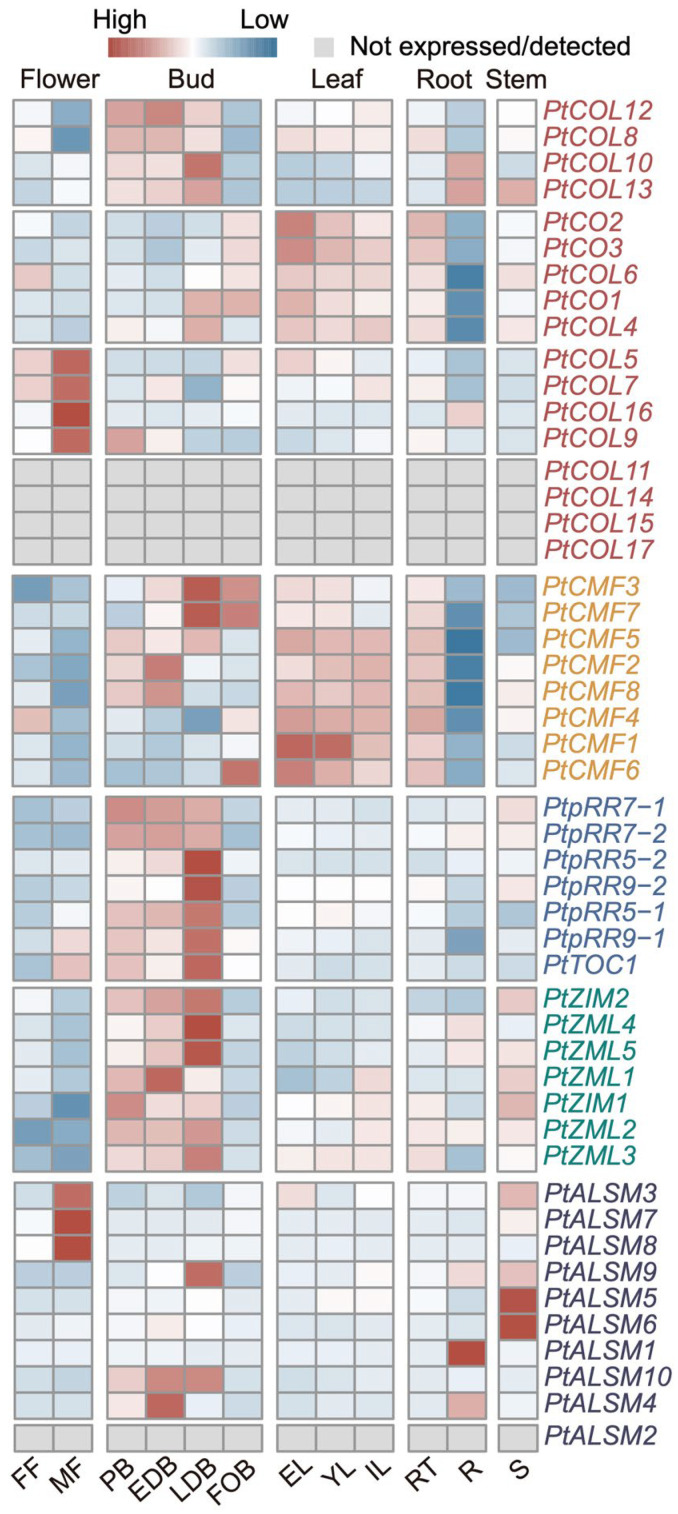
Expression patterns of *PtCCT* genes across various tissues. The tissue-specific expression profiles of *PtCCT* genes were examined in a range of anatomical structures, including female flower (FF), male flower (MF), pre-dormant bud (PD), early dormant bud (EDB), late dormant bud (LDB), fully open buds, fully expanded leaves (EL), young leaves (YL), immature leaves (IL), root tips (RT), roots (R), and stems (S). The RNA-seq expression data were downloaded from the publicly available Phytomine database.

To delineate the distinctive tissue-specific expression profiles of *PtCCT* genes, we accessed RNA expression data derived from *Populus*, sourced from the Phytomine database (Table S3). In total, 13 out of 17 *PtCOLs* exhibited detectable expression across diverse tissues and were amenable to categorization into three well-defined clusters based on their expression patterns. Specifically, *PtCOL8*, *PtCOL10*, *PtCOL12*, and *PtCOL13* displayed predominant expression in dormant buds (PB, EDB, and LDB); *PtCOL1*, *PtCOL2*, *PtCOL3*, *PtCOL4*, and *PtCOL6* exhibited elevated expression levels in leaves (EL, YL, and IL), as well as in root tissues (RT); and *PtCOL5*, *PtCOL7*, *PtCOL9*, and *PtCOL16* demonstrated prominent expression in male flowers (MF) (Figure 3). Notably, nearly all *PtCMF* genes displayed heightened expression levels in leaves and root tissues, with *PtCMF2*, *PtCMF3*, *PtCMF5*, *PtCMF7*, and *PtCMF8* also exhibiting expression in buds. Of particular interest, all *PtPRR* and *PtZIM* genes exhibited distinctive expression profiles primarily within dormant buds (PB, EDB, and LDB), implying their potential pivotal role in the regulation of bud dormancy in *Populus*. Conversely, within the ALSM clade, these genes displayed dispersed expression across various tissues, suggesting a diverse range of functions for *PtALSM genes*. Given the prominent expression of PtPRRs and several PtCOLs in dormant buds, we next examined whether members of the CCT family also displayed coordinated seasonal expression dynamics in leaves and shoot apices.

### 3.4. Seasonal Transcriptome Dynamics of PtCCTs in Leaf and SA

**Figure 4:**
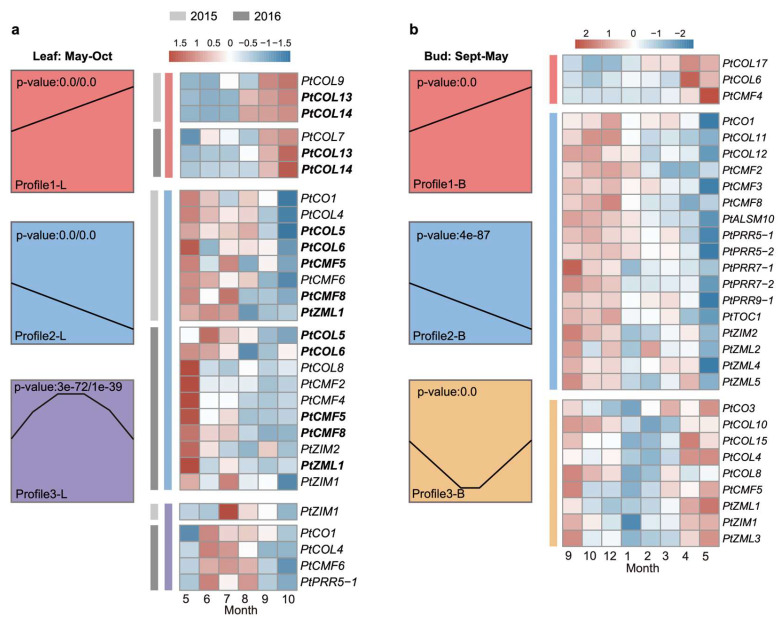
Seasonal expression patterns of *PtCCT* genes in leaves and buds. The investigation of seasonal gene expression patterns in *PtCCT* genes was conducted through the application of the Short Time-series Expression Miner (STEM) analysis, coupled with the generation of heatmaps for each noteworthy expression profile. (**a**) In the context of leaves, three distinct and significant expression profiles extending from May to October were identified and assigned unique colors: Profile1-L (red), Profile2-L (blue), and Profile3-L (purple). The shading in light and dark gray corresponds to the gene expression levels observed in the years 2015 and 2016, respectively. Genes highlighted within the heatmap exemplify stable expression patterns that persisted across the two-year timeframe. (**b**) For bud samples taken from September to May, STEM analysis revealed three prominent and significant expression profiles, each designated by a distinct color: Profile1-B (red), Profile2-B (blue), and Profile3-B (yellow).

This study encompassed the investigation of seasonal expression patterns in leaves, spanning from May to October, representing the phases of leaf growth to senescence, as well as the SA, which extended from September to May, encapsulating the transition from bud dormancy to dormancy release. Our analysis revealed the presence of three distinct and significant expression profiles within the seasonal transcriptomes of leaves and SA (Figure 4a,b). These profiles were identified using STEM, and only model profiles with *p* < 0.05 were considered significant. In the context of leaf expression, 12 *PtCCTs* exhibited significant expression profiles in 2015, and 17 *PtCCTs* demonstrated the same in 2016, as detailed in Tables S4 and S5. In Profile1-L, *PtCOL13* and *PtCOL14* exhibited a stable expression pattern, with their highest expression levels recorded in September and October for both years. In Profile2-L, *PtCOL5*, *PtCOL6*, *PtCMF5*, *PtCMF8*, and *PtZML1* displayed a consistent downregulated pattern, with peak expression levels occurring in May. However, we did not identify any genes displaying stable expression patterns in Profile3-L across the two years, suggesting that these genes may be more susceptible to short-term environmental fluctuations.

Regarding SA expression, three *PtCCT* genes (*PtCOL6*, *PtCOL17*, and *PtCMF4*), characterized by their highest expression in April and May during the active growth phase, were assigned to Profile1-B (Figure 4b). In total, 17 *PtCCT* genes, including *PtCO1*, *PtCOL11*, *PtCOL12*, *PtCMF2*, *PtCMF3*, *PtCMF8*, *PtALSM10*, six *PtPRR* genes, *PtZIM2*, and *PtZML2*, *PtZML4*, and *PtZML5*, exhibited increasing expression trends from September to December and were categorized under Profile2-B. In contrast, *PtCO3*, *PtCOL4*, *PtCOL8*, *PtCOL10*, *PtCOL15*, *PtCMF5*, *PtZIM1*, *PtZML1*, and *PtZML3* were characterized by downregulated expression during the winter dormancy period and were grouped within Profile3-B. Notably, nearly all genes within the PRR clade were assigned to Profile2-B, signifying their heightened expression levels during the period from September to December, aligning with the induction of growth cessation under short-day conditions. Detailed seasonal expression data for *PtCCTs* in SA are available in Table S6. Because seasonal regulation in perennial trees is closely linked to photoperiodic and circadian control, we next examined whether *PtCCT* genes also exhibited diurnal rhythmic expression.

### 3.5. The Diurnal Rhythmic Expression Analysis of PtCCTs

**Figure 5:**
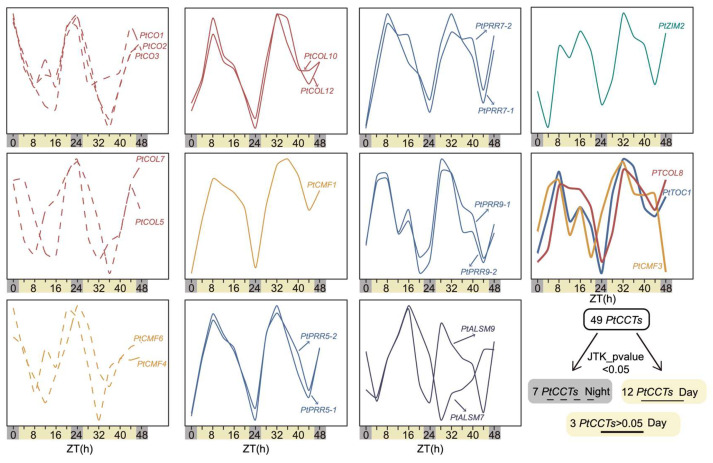
Diurnal dynamic expression patterns of *PtCCT* genes. The diurnal dynamics expression profiles of *PtCCT* family genes under long photoperiods (light:dark [LD] 16 h:8 h, 48 h). The diurnal expression matrix (light:dark [LD] 16 h:8 h, 48 h) was obtained from Edwards’s research of hybrid aspen [39]. The JTK_CYCLE methodology was employed to identify *PtCCT* genes exhibiting statistically significant diurnal rhythms (JTK_pvalue < 0.05). A total of 19 *PtCCT* genes were found to display significant rhythmic expression patterns, and they were categorized into two groups: 7 *PtCCT* genes demonstrated peak expression during the nighttime (dotted lines), while 12 *PtCCT* genes exhibited a tendency to peak during the daytime (solid lines). Notably, diurnal dynamics were also observed in *PtCOL8*, *PtCMF3*, and *PtTOC1*, despite their JTK_pvalues exceeding 0.05, as indicated by the thicker lines.

We employed the JTK_Cycle method to identify genes exhibiting significant diurnal rhythmicity, as indicated by a JTK_pvalue < 0.05. Subsequently, we assessed the phase of peak expression for CCT family genes in *Populus*. In total, 19 *PtCCT* genes exhibited noteworthy diurnal rhythmic expression patterns, and they could be categorized into two distinct groups based on their peak expression periods. Specifically, *PtCO1*, *PtCO2*, *PtCO3*, *PtCOL5*, *PtCOL7*, *PtCMF4*, and *PtCMF6* demonstrated heightened expression during the nighttime hours. In contrast, *PtCOL10*, *PtCOL12*, *PtCMF1*, *PtALSM7*, *PtALSM9*, six *PtPRR* genes (*PtPRR5*-1, *PtPRR5*-2, *PtPRR7-1*, *PtPRR7-2*, *PtPRR9-1*, and *PtPRR9-2*), and *PtZIM2* exhibited peak expression during the daytime (Figure 5 and Table S7). It is noteworthy that while the JTK_pvalue exceeded 0.05 in certain genes, they still displayed discernible diurnal dynamics. This observation applied to *PtCOL8*, *PtCMF3*, and *PtTOC1* (Figure 5). Intriguingly, *PtCOLs* belonging to distinct clades exhibited varying diurnal rhythms: *PtCO1*, *PtCO2*, *PtCO3*, *PtCOL5*, and *PtCOL7*, which formed COL-Clade I, exhibited peak expression during the nighttime, while those in COL-Clade II (*PtCOL8*, *PtCOL10*, and *PtCOL12*) demonstrated peak expression during the daytime. Among the rhythmically expressed genes, PtPRRs were particularly notable because they combined strong daytime oscillation with pronounced seasonal upregulation during the growth-cessation period; therefore, we further analyzed *PtPRRs* in relation to photoperiodic signaling.

### 3.6. Expression Dynamics of PtPRRs in Relation to Photoperiodic Signaling

**Figure 6:**
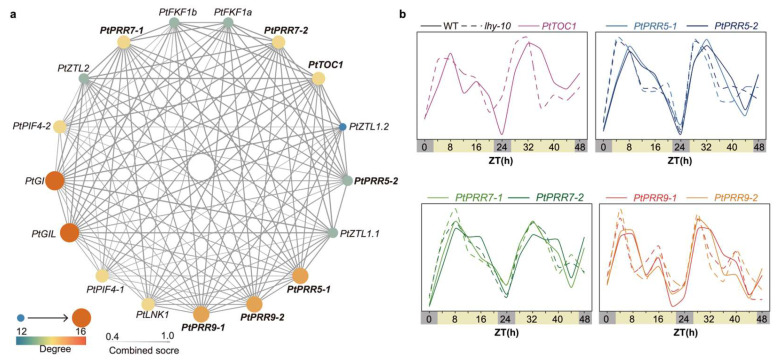
Predicted interactions of *PtPRRs* with photoperiod pathway components and their diurnal expression in wild-type and lhy-10 plants. (**a**) STRING-based protein–protein interaction network of PtPRRs and core photoperiod/circadian regulators in *Populus*. Node color indicates degree (number of connections), and edge thickness reflects the combined interaction score. (**b**) Diurnal expression profiles of *PtTOC1*, *PtPRR5-1/2*, *PtPRR7-1/2*, and *PtPRR9-1/2* in wild-type (WT, solid lines) and *lhy-10* RNAi plants (dashed lines). The x-axis indicates zeitgeber time (ZT, h).

Because *PtPRRs* were distinguished by both significant diurnal rhythmicity and strong seasonal upregulation in dormant tissues, we next examined their potential links to photoperiodic signaling. The functional and physical interactions of the seven *PtPRRs* were investigated using STRING. The results revealed that *PtPRRs* showed interaction with a collection of photoperiodic pathway genes (Figure 6a), such as *PtGI* (*GIGANTEA*), which plays an important role in growth cessation and bud set in *Populus* [42]. Within this investigation, we were able to discern distinctive expression patterns in *PtPRRs*, with a notable emphasis on their pronounced expression within dormant buds and their heightened expression during the period from September to December, coinciding with the growth cessation induced by short-day (SD) conditions (Figure 4b and Figure 5). This finding leads us to hypothesize that *PtPRRs* may serve a pivotal role in the SD-induced growth cessation of *Populus*. In the context of *Populus*, it has been established that *LATE ELONGATED HYPOCOTYL 2* (*LHY2*) operates to suppress the expression of *FT2* under SD conditions. The downregulation of *FT2*, in turn, accelerates the process of growth cessation, as substantiated by prior research [4,43,44]. In our study, we further assessed the diurnal expression patterns of *PtPRRs* in wild-type hybrid aspen (*P. tremula* × *P. tremuloides*, clone T89) and *lhy-10* RNAi lines, where the expression of *LHY1* and *LHY2* was reduced by approximately 40%. The experimental data, encompassing diurnal expression profiles, were obtained from the previous study [39]. Notably, in the *lhy-10* plants, a phase shift in the peak expression was observed, occurring earlier in comparison to the WT, specifically for *PtPRR5-1/2*, *PtPRR9-1/2*, and *PtTOC1*. Additionally, *lhy-10* plants exhibited higher peak expression levels for *PtPRR7-1/2* (Figure 6b). Together, these results indicate that *PtPRRs* act as circadian output components that are sensitive to *LHY* activity and are likely to contribute to the photoperiodic regulation of growth cessation and bud set in *Populus*.

## 4. Discussion

### 4.1. Phylogenetic Analysis and Evolution of PtCCTs

The CCT family genes, which take part in regulating flowering, circadian rhythms, development, and abiotic stress tolerance [17], have undergone extensive investigations in multiple plants. However, the temporal deployment and potential roles of the CCT family in seasonal dormancy and circadian regulation in Populus remain insufficiently understood. In this study, we systematically identified and classified 49 putative *PtCCTs* within the *Populus* genome, categorizing them into five distinct subfamilies, namely COL, CMF, PRR, ALSM, and ZIM, based on a combination of domain compositions and phylogenetic relationships. Notably, our findings align with prior analyses of CCT family genes in other plant species [45], underscoring the conservation of this phylogenetic distribution across various plant taxa. The overall phylogenetic organization of *PtCCTs* is also broadly consistent with that reported in other angiosperms, including Arabidopsis, rice, pear, foxtail millet and soybean, in which COL-, PRR-, and CMF-related groups are similarly conserved [45,46,47]. This suggests that the basic framework of the CCT family was established early during angiosperm evolution, whereas lineage-specific expansion may have contributed to functional diversification in different plant groups. Our investigation revealed that the COL subfamily comprised the largest number of CCT genes within *Populus*, accounting for 34.7% (17 *PtCOLs*) of the total *PtCCTs* (Figure 2). Furthermore, this subfamily exhibited a subdivision into two distinct subclades. It is worth noting that our results differ from a previous study that identified 14 COL genes in Populus based on the presence of conserved domains, including both B-box and CCT domains [8]. The outcomes of our analysis, considering both gene domain compositions and the phylogenetic relationships of CCT family genes across *Populus*, *Arabidopsis*, and rice, have provided a more comprehensive and refined perspective, advancing our understanding of this study. Previous studies have provided an important foundation for understanding CCT genes in poplar. A recent genome-wide analysis identified CCT transcription factors in poplar and described their chromosomal distribution, synteny relationships, promoter cis-elements, and expression features [26]. In addition, functional analysis of *PpnCCT39* showed that this gene supports chlorophyll biosynthesis and photosynthesis in poplar [48]. Compared with these studies, the novelty of our work lies not only in an expanded and refined characterization of the *Populus* CCT family but more importantly in the integration of tissue-specific, seasonal, and diurnal transcriptome datasets to examine the temporal deployment of CCT genes in perennial tissues. Our analyses therefore move beyond structural annotation and single-gene functional description and instead provide a family-wide framework linking *PtCCTs*, especially *PtPRRs* and specific *PtCOLs*, to circadian regulation, seasonal growth cessation, and bud dormancy in *Populus*. Thus, unlike previous poplar studies that mainly emphasized gene family composition or the physiological function of a single member, our study addresses how CCT genes may be deployed across perennial tissues during seasonal transitions and daily rhythmic cycles.

WGD stands as a pivotal driver in the evolutionary trajectory of plants, with at least two such events being recurrent and conserved in the evolutionary history of all angiosperms [49,50]. Furthermore, WGD serves as a crucial mechanism contributing to the amplification of gene families, thereby propelling the emergence of novel functionalities in genome evolution [34,51,52]. Within the cohort of identified *PtCCTs*, it is noteworthy that a total of 21 gene pairs originated through segmental duplication, whereas only one gene pair arose through tandem duplication. This pattern strongly suggests that the expansion of CCT family genes in *Populus* primarily stems from segmental duplication events. These findings are consistent with analogous outcomes observed in recent investigations of CCT family genes in various plant species, including *Foxtail millet*, *Pyrus bretschneideri*, and *Glycine max* [45,46,47]. Moreover, the observed ranges of *Ks* within duplicated genes align with the timings of the two WGD events, known as the *p* and γ events, within the *Populus* genome [53]. The *Ks* values provide insights into the evolutionary timescales, indicating that 16 gene pairs originated from the *p*-WGD event, displaying *Ks* values within the range of 0.21 to 0.37 (11.32–20.1 mya). Additionally, five gene pairs trace their origins to the γ-WGD event, characterized by *Ks* values ranging from 1.50 to 2.34 (82.38–128.50 mya). Furthermore, the *Ka*/*Ks* ratio, which spans a range from 0.1187 to 0.4392, with a mean value of 0.2808, underscores the prevalence of purifying selection acting upon these duplicated genes. These results suggest that, following duplication, most PtCCT genes have been maintained under functional constraint, while some duplicated members may still have undergone divergence in expression or regulatory context.

### 4.2. Potential Involvement of PtPRR and PtCOL Genes in Light Signaling

Light signaling constitutes a pivotal environmental determinant encompassing facets such as light quality, intensity, and photoperiod, exerting profound regulatory effects on plant growth and developmental processes [54]. Notably, seasonal fluctuations in photoperiod represent a primary environmental cue governing seasonal growth, the dormancy cycle, and flowering timing in perennial plants [20,37]. Transcriptional regulation and gene functionalities are intricately linked with the presence of cis-regulatory elements within promoter regions [55]. Hence, we undertook the prediction of cis-regulatory elements within the *PtCCT* genes by analyzing 2-kilobase upstream sequences of coding sequences, employing the PlantCARE web database. Our analysis unveiled an enrichment of G-box and ABRE elements in the promoter regions of *PtPRRs* and *PtCOLs* within the COL-Clade I (Figure S1). Notably, the G-box DNA sequence motif maintains a high degree of conservation across plant species and serves as a common component within promoters of light-regulated genes [56]. Previous research has established the specific binding of the basic helix–loop–helix (bHLH) transcription factor PIF3 to the G-box motif. Furthermore, it has been demonstrated that phytochrome B (*PHYB*) can engage in reversible binding interactions with G-box-bound *PIF3* in *Arabidopsis* [57]. The PHYB-PIF8 regulon in *Populus* has been substantiated to exercise control over seasonal growth [58]. Similar associations between light-responsive cis-elements and photoperiodic regulators have been widely documented in model plants, whereas in woody perennials these regulatory relationships are further embedded in seasonal growth control. Therefore, the enrichment of G-box and ABRE motifs in *PtPRRs* and specific *PtCOLs* is consistent with, but does not by itself demonstrate, their involvement in light- and hormone-related transcriptional regulation during seasonal transitions.

In addition, it is noteworthy that the abscisic acid (ABA) pathway plays a significant role in short-day (SD)-induced bud dormancy in *Populus* [21]. Our findings suggest that PtPRRs and PtCOLs, particularly those showing significant diurnal rhythmicity (JTK_pvalue < 0.05; Figure 5), may be associated with photoperiod-related growth cessation through transcriptional networks involving G-box- and ABRE-binding factors. However, these putative associations remain to be experimentally validated. Furthermore, our results show that *PtPRRs* are distinctly expressed in dormant buds, displaying conspicuous circadian rhythmic patterns with peak expression during the daytime, while also being expressed during the period spanning September to December, coinciding with the bud set stage (Figure 3 and Figure 4). Taken together, these observations are consistent with the possibility that PtPRRs participate in photoperiod-related regulation of growth cessation and bud set in Populus. However, because this inference is based mainly on cis-element prediction and transcriptome patterns, direct functional evidence is still required.

### 4.3. PtPRRs in Relation to the Circadian Clock and Seasonal Growth Cessation in Populus

In *Arabidopsis*, *PRR* genes participate in transcriptional–translational feedback loops in which they repress the morning clock components *CIRCADIAN CLOCK ASSOCIATED 1* (*CCA1*) and *LHY* [59]. At the same time, the expression of *PRR9* and *PRR7* is activated by *CCA1* and *LHY*, whereas *TOC1* and possibly *PRR5* are repressed by these morning factors [60,61]. In Populus, *PtPRR5* has been shown to directly regulate genes encoding transcription factors involved in the control of flowering time, indicating an important role in the reproductive phenology of this woody species [62]. Interestingly, while *PRR5* promotes flowering in Arabidopsis, its overexpression in rice delays flowering, suggesting that PRR5-like genes can exert distinct effects on developmental timing in different species [62]. In hybrid aspen, growth cessation is controlled by the circadian clock and involves the molecular circuitry formed by *PttLHY1*, *PttLHY2* and *PttTOC1* [25]. Taken together, these studies in model plants and woody perennials suggest that PRR genes are conserved clock-associated components but that their downstream developmental outputs may vary among species and life-history strategies.

In our study, several observations point to a close relationship between *PtPRRs*, the circadian clock, and short-day (SD) responses in *Populus*. First, STRING analysis indicated that *PtPRRs* interact with photoperiodic pathway components such as *PtGI*, and many *PtPRRs* showed high expression in dormant buds and during the period from September to December, when trees undergo SD-induced growth cessation. Second, in lhy-10 RNAi plants, in which *LHY1* and *LHY2* expression is reduced by ~40%, the diurnal expression profiles of several *PtPRRs* were phase-advanced and/or showed altered peak amplitudes compared with wild-type plants, suggesting that PtPRRs may respond to changes in LHY activity and may be associated with circadian clock outputs.

These findings are consistent with a scenario in which *PtPRRs* may act as circadian output components that respond to SD-induced changes in the clock and are transcriptionally active during the period of growth cessation and bud set. However, our current data are correlative and do not yet demonstrate a direct causal role of individual *PtPRRs* in controlling SD-induced growth cessation. Unlike functional studies in *Arabidopsis*, rice, or hybrid aspen, the conclusions presented here are based primarily on transcriptome dynamics, promoter composition and interaction network inference. Therefore, the proposed roles of *PtPRRs* in seasonal growth cessation should be regarded as testable hypotheses rather than direct functional demonstrations. Future studies based on targeted knockouts, overexpression lines or other genetic approaches will be required to determine whether specific *PtPRRs* can directly modulate the timing of growth cessation and whether they can serve as reliable molecular markers of bud dormancy depth in *Populus*.

## 5. Conclusions

This study represents a comprehensive exploration of the CCT family genes in *Populus*. The investigation led to the identification of a total of 49 CCT family genes within the *Populus* genome, which were subsequently categorized into five subfamilies based on the examination of their phylogenetic relationships and conserved domains. This categorization was instrumental in unveiling the evolutionary characteristics underpinning the *PtCCT* genes. Additionally, the expression analysis conducted in this study shed light on the multifaceted functions carried out by *PtCCTs*, with some members demonstrating pronounced circadian rhythmic and seasonal expression profiles. This study suggests that *PtPRRs* are promising candidates associated with seasonal growth cessation under SD conditions.

## Data Availability

Data will be made available on request.

## Supplementary Materials

The following supporting information can be downloaded at: https://www.mdpi.com/article/10.3390/genes17030346/s1.
