# Supplementary material for: Genome-Wide Characterization and Seasonal–Circadian Expression Analysis of CCT Family Genes in Populus
Source: Genes (Basel). 2026 Mar 20;17(3):346. doi: 10.3390/genes17030346 (PMC13026854; doi:10.3390/genes17030346)
Supplement: Supplementary file 1 [file genes-17-00346-s001.zip › supplement_figure.pdf]

Genome-Wide Characterization and Seasonal–Circadian Expression Analysis of CCT Family Genes in *Populus*

Rui Zang [1], Yue Li [2] and Xiaokang Dai [1,2,*]

[1] Engineering Research Center of Agricultural Microbiology Technology, Ministry of Education & Heilongjiang Provincial Key Laboratory of Ecological Restoration and Resource Utilization for Cold Region & Key Laboratory of Microbiology, College of Heilongjiang Province & School of Life Sciences, Heilongjiang University, Harbin 150080, China
[2] Hubei Hongshan Laboratory, Hubei Engineering Technology Research Center for Forestry Information, College of Horticulture and Forestry Sciences, Huazhong Agricultural University, Wuhan 430070, China
* Correspondence: daixiaokang@hlju.edu.cn

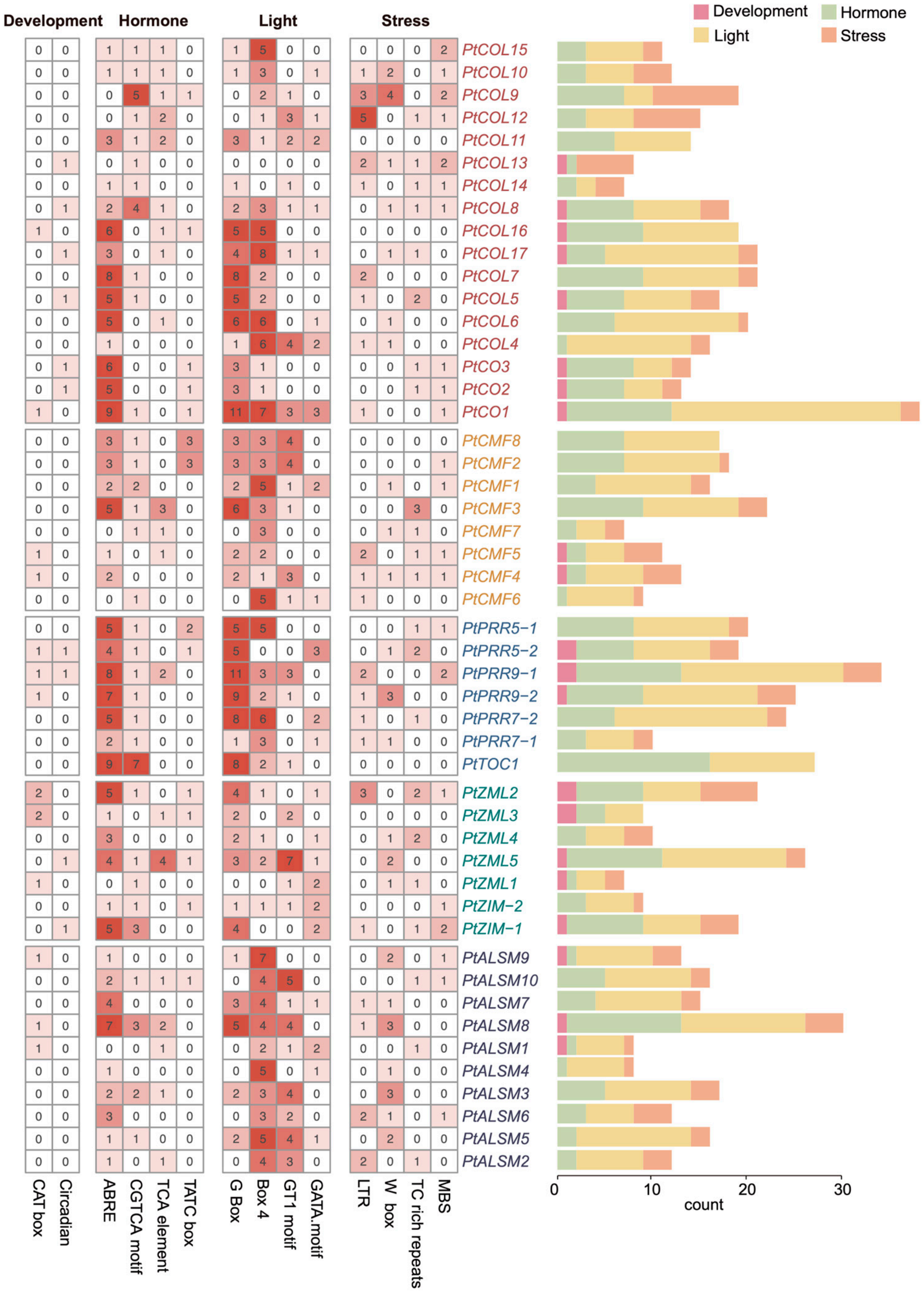

Figure S1 Distribution of cis-acting elements within the promoter sequences of *PtCCTs* genes. The 2000 base pair upstream sequences of *PtCCTs* genes were computationally predicted using the PlantCARE database. The heatmap illustrates the quantity of cis-acting elements present within the promoters of *PtCCTs* genes, while the accompanying bar plot conveys the respective counts for each category of cis-element.

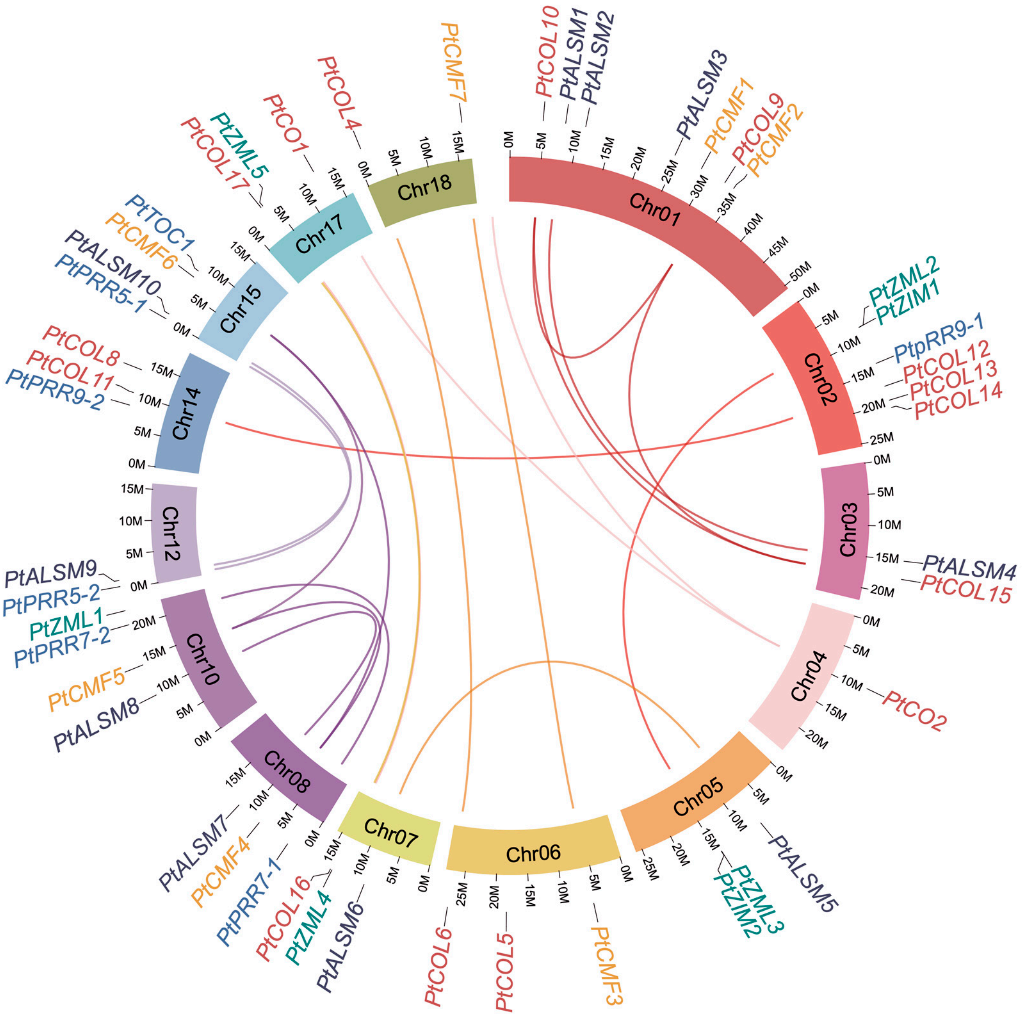

Figure S2 Schematic depictions of collinear associations among *Populus* CCT family genes. Collinearity and gene duplication analyses of the CCT family genes were executed using the MCScanX software. The resulting syntenic relationships were represented through visualization employing Circos version 0.69. Chromosomes of *Populus* are shown in different colors and the lines in the center show the syntenic gene sets.
